# X chromosome inactivation: new players in the initiation of gene silencing

**DOI:** 10.12688/f1000research.10707.1

**Published:** 2017-03-27

**Authors:** Ines Pinheiro, Edith Heard

**Affiliations:** 1 Mammalian Developmental Epigenetics Group (équipe labellisée La Ligue), Institut Curie, PSL Research University, CNRS UMR3215, INSERM U934, 26 Rue d’Ulm, 11 75248 Paris Cedex 05, France

**Keywords:** X chromosome inactivation, XCI, Xist RNA, X-linked genes, chromatin, epigenetics

## Abstract

X chromosome inactivation (XCI) is a dosage compensation process that was adopted by female mammals to balance gene dosage between XX females and XY males. XCI starts with the upregulation of the non-coding RNA Xist, after which most X-linked genes are silenced and acquire a repressive chromatin state. Even though the chromatin marks of the inactive X have been fairly well described, the mechanisms responsible for the initiation of XCI remain largely unknown. In this review, we discuss recent developments that revealed unexpected factors playing a role in XCI and that might be of crucial importance to understand the mechanisms responsible for the very first steps of this chromosome-wide gene-silencing event.

## Introduction

In many animal species, males and females have unbalanced genetic content regarding the sex chromosomes (reviewed in 1). In mammals, females have two X chromosomes and males have an X and a Y chromosome. Sex chromosomes originated from autosomes. The X chromosome retained >95% of its ancestral genes, while the Y chromosome retained only 2% of its ancestral genes ^2, 3^. This generates a genetic imbalance for ancestral genes between males and females, for which various dosage compensation strategies have evolved across different species. In mammals, females transcriptionally inactivate one of their two X chromosomes in a process called X chromosome inactivation (XCI) ^1, 4^.

In mice, XCI occurs in two waves during early development. A first wave of XCI starts between the 4–8-cell stage ^5^. This results in imprinted inactivation of the paternal X chromosome (Xp) ^6, 7^. The Xp is then reactivated at the blastocyst stage and random XCI occurs, implying that either the maternal X chromosome (Xm) or the Xp is silenced ^8^. After this choice is made, the inactive state is inherited throughout cell division. XCI begins with expression of the long non-coding RNA (lncRNA) Xist from the chromosome that will be silenced ^9^. Xist RNA coats the X chromosome in *cis*, resulting first in rapid depletion of RNA polymerase II (RNA Pol II) and euchromatic histone marks (such as histone acetylation) ^10^. Xist RNA-coated chromatin then becomes enriched in repressive histone ubiquitination and methylation marks (H2AK119ub and H3K27me3) ^5, 11, 12^. In addition, Xist RNA coating triggers spatial reorganization of the X chromosome ^13, 14^.

Chromatin seems an obvious candidate as the template targeted by Xist RNA to carry the *cis*-limited epigenetic memory of the X chromosome during XCI. The chromatin modifications that decorate the inactive X (Xi) and the kinetics of their deposition have been well defined using immunofluorescence during embryonic stem cell (ESC) differentiation or early embryogenesis ^5, 11^ or chromatin immunoprecipitation for candidate histone modifications followed by qPCR for target genomic regions ^15^. Despite several decades of research, the transcription- or chromatin-associated mechanisms responsible for the initiation of *Xist*-mediated gene silencing have remained unclear.

Recently, three different groups developed methods that allow the pull down of endogenous Xist and its associated proteins ^16– 18^. These approaches identified factors that might have important roles during the initiation of XCI. Highlighting their roles, two genetic screens for factors involved in the onset of Xist-mediated gene silencing (as opposed to its maintenance ^19^) identified the same proteins ^20, 21^.

In this review, we will discuss these exciting new discoveries as well as some of the open questions regarding Xist-mediated gene repression during XCI.

## The spatiotemporal dynamics of Xist RNA association, gene silencing, and escape

XCI is a dynamic process operating at different levels, including gene silencing, chromatin modifications, and chromosome reorganization (reviewed in 22). Upon *Xist* expression, genes along the Xi start to be progressively silenced, with groups of genes that are silenced at early, mid, or late stages during XCI ^23^. The reasons why the kinetics of gene silencing vary so dramatically from one gene to another are not clear. It has been suggested that silencing might progress as a linear gradient from the *Xist* gene within the X-inactivation center (Xic) ^24^, with the genes closest to the Xic being silenced first. However, although some genes close to the Xic are silenced early, others are not ^23^. In fact, the spread of XCI should be considered in the context of the 3D chromosome organization of the X, whereby distant genomic regions can come in close contact because of chromosome folding ^25^. This was investigated using RNA-antisense purification (RAP) that allows the purification of *Xist* RNA–DNA contacts ^26^. By sequencing the RNA-bound DNA, the chromosome contact regions of Xist RNA were mapped during XCI, revealing that the earliest regions associated with Xist were those that came into closest proximity to the Xic region based on chromosome conformation capture (HiC) data ^27^. Similar results were found using a capture hybridization analysis of RNA targets with deep sequencing, or CHART-seq ^28^. Both studies further revealed that Xist RNA tends to initially bind in close proximity to active gene-rich regions, correlating with sites of H3K27me3. How do these findings relate to gene silencing kinetics? Although the ESC systems involving doxycycline induction of *Xist* used in these studies undergo more synchronized XCI, this might not entirely reflect the normal kinetics of XCI. To address this question *in vivo*, a recent study examined the X-chromosome-wide gene-silencing kinetics during imprinted XCI, a system with endogenous initiation of XCI that is triggered by paternal Xist expression from the 2–4-cell stage. Single-cell RNA-seq of single blastomeres from F1 hybrid mouse embryos at every stage of pre-implantation development revealed that many of the genes silenced earliest in embryos lie within the first *Xist*-bound regions as defined in ESCs. However, some late-silenced genes, or even escapees, also lie in their vicinity ^29^. Similar findings were reported in hybrid mouse ESCs during differentiation towards embryoid bodies and neural progenitor cells ^30^. This implies that although 3D proximity to *Xist* indeed seems to promote XCI of early silenced genes, this is not the only factor predicting the silencing kinetics of any particular region. Whether there is local spread in *cis* from Xist-coated regions and whether this relates to the local chromatin context or proximity of certain regions to the nuclear envelope remains to be found.

## Escape from X inactivation

How some genes avoid XCI altogether is another interesting question. Several such constitutive “escapees” exist in mice ^31^ and even more in humans ^32^. Indeed, in both the RAP and the CHART-seq studies, the constitutive escapee *Jarid1c* was found to be surrounded by Xist-binding sites that are abruptly depleted at this locus. This observation implies that there must be some features at the *Jarid1c* genomic locus (and the factors that bind to it) or else in its chromatin structure that prevent Xist RNA from spreading into this region. The sequences responsible for escape from gene silencing remain to be identified. Transgenesis studies have shown that *Jarid1c* has an intrinsic capacity to escape, with *Jarid1c* transgenes being capable of escape from XCI at different locations on the X chromosome ^33^. More detailed BAC integration studies mapped elements required for escape to the region upstream or within the *Jarid1c* gene ^34^. A candidate sequence-specific escape-promoting factor that has been proposed is CTCF (CCCTC-binding factor) ^35^. CTCF is a zinc-finger DNA-binding protein that has been identified as an insulator between different genomic regions ^36^, maintaining a gene in a spatial functional domain with its own regulatory elements ^37^. CTCF is widely bound across the mouse and human genomes ^38^. It is also bound to the X chromosome, including transcription start sites (TSS) of escapees ^39^, suggesting a possible role for the transcriptional state of these genes. In fact, escape from Xist-mediated gene silencing correlates with the presence of CTCF at the TSS of escaping genes on the X chromosome, but not when a *Xist* transgene is integrated in an autosome (Loda *et al*., unpublished data). More detailed analysis of the possible role of the CTCF-binding sites flanking the *Jarid1c* locus is still intriguing ^35^. On the one hand, deleting 3’ CTCF sites does not prevent *Jarid1c*’s capacity to escape. Rather, this promotes illegitimate escape of neighboring genes. On the other hand, flanking GFP transgenes inserted in the *Hprt* locus with CTCF-binding sites did not prevent its silencing upon *Xist* expression ^40^. Taken together, these data suggest that CTCF might not be sufficient to explain escape. It would be interesting to know whether there is a common mechanism of escape (do other escapees display similar behavior when inserted into different X-linked regions?) and whether there are common genomic or epigenetic features or trans-factors involved.

In addition to genes that constitutively escape from XCI, tissue-specific escapees that are silenced during XCI in pre-implantation embryos and become reactivated in certain tissues also exist. Whether Xist-binding sites are found in the vicinity of such loci when they are silenced but then disappear in the tissues in which they escape is not yet known. The nature of the regulatory regions and epigenomic features of these escapees was recently hinted at based on allele-specific ATAC seq ^39^. The mechanisms allowing such regions to overcome the heterochromatic state and become reactivated remain unclear. In the environment of the silent X, where many chromatin modifiers play a role in locking in a very stable silent state, it will be important to understand the features of regions that can revert from this repressive state and the extent to which this is driven by specific transcription factors, chromatin states, chromosome architectural proteins, or nuclear compartments.

## Gene silencing during X-chromosome inactivation: mechanisms and key players

As mentioned above, *Xist* expression is accompanied by global changes in chromatin and gene expression. Genetic dissection of the Xist transcript revealed that a highly conserved region called the A-repeat (on exon 1) ^41^ is necessary for Xist’s gene-silencing role during XCI ^42^. However, the mechanisms responsible for gene silencing and the importance of chromatin-modifying complexes for the initiation of gene silencing are still largely unknown. Most of the studies characterizing Xi chromatin were based on indirect immunofluorescence approaches, which are quite crude assays and might highlight only the players that are more enriched in the Xi territory. Additionally, it was observed that the exclusion of RNA Pol II and histone acetyl marks from the Xi territory precedes gene silencing, indicating that there might be other protein complexes important for the initiation of X-linked gene silencing.

One of the obvious first steps needed in order to learn about the mechanisms involved in Xist-mediated gene silencing is the identification of the protein, RNA, and DNA partners of Xist RNA that likely mediate its functions. This represented a technical challenge for many years, as Xist RNA pull down experiments present many difficulties and only recently have systematic approaches yielded conclusive data ^16– 18^. One reason for this was that RNA pull downs often retrieve many non-specific interacting proteins that bind nucleic acids in general. Another challenge was the length of the Xist RNA molecule (>17,000 nucleotides), which renders it difficult to manipulate and to use for capture while maintaining its integrity.

In the past year, several groups have finally been able to isolate factors that bind Xist RNA specifically using a variety of biochemical approaches that rely on the purification of Xist and its binding partners followed by mass spectrometry ^16– 18^. Many of these have turned out to be important for the initiation of gene silencing during XCI. Furthermore, genetic screens were applied to identify factors that impair Xist-mediated gene silencing ^20, 21^.

## Factors implicated in the initiation of gene silencing during X-chromosome inactivation

Thanks to these two types of approach, a set of factors that are necessary for the initiation of Xist-mediated gene silencing has been defined. Amongst these different methods and the different lists of candidate proteins identified, one factor identified in all studies was Spen (for “Split-ends”, which was originally identified in *Drosophila melanogaster* ^43^). Spen is a very large protein with several RNA-binding domains. It has been implicated in transcriptional silencing owing to its interaction with the NuRD-MBD3 complex, more particularly via HDACs 1 and 2 ^44, 45^ or HDAC3 ^46^. Spen is proposed to facilitate the initiation of XCI through direct binding to the Xist RNA A-repeat, via its RNA-binding motifs, and by recruitment of HDACs to the future Xi. siRNA-mediated knockdown of HDAC3, but not of HDAC1 or 2, phenocopied the results obtained with Spen knockdown ^17^. In the absence of Spen or HDAC3, *Xist* RNA coating is normal but is reported to no longer lead to RNA Pol II depletion or Ezh2 enrichment and to result in defective gene silencing. Given its huge size, Spen might interact with multiple different proteins in various complexes. Although a spectrum of Spen-interacting proteins has previously been described ^46^, a Spen-XCI-specific protein complex has not yet been characterized.

## Factors associated with PRC1 and 2 complexes

Another revelation from these studies was the identification of PRC1 but not PRC2 members as Xist-interacting proteins ^16, 18^. This appears to challenge the prevailing view that PRC2 is directly recruited by Xist. In the study by the Lee lab ^18^, the PRC1 factor RING1 was identified. The Chang lab identified RING2, RYBP, and PCGF5 ^16^. Indeed, the latter factor is one of those that distinguish non-canonical PRC1, the variant of PRC1 that is directly targeted to chromatin independently of PRC2 or H3K27me3 ^47, 48^. Thus, it appears that Xist-mediated Polycomb recruitment may not follow the model for hierarchical PRC2-PRC1 recruitment ^49^. A recent study supports this order of events: initial Xist-mediated PRC1 recruitment followed by indirect recruitment of PRC2 through Jarid2 ^50^. Indeed, Jarid2 may bind to PRC1-mediated H2AK119Ub, thus enabling PRC2 to become recruited. However, the situation may be complex, with multiple parallel pathways for PRC2 and PRC1 recruitment to the Xi ^51^.

In fact, like PRC2-H3K27me3, PRC1-H2AK119Ub had already been implicated in an early time-window during XCI ^52, 53^. Differentiation of Eed ^–/–^ ESCs revealed that, upon Xist induction, the region corresponding to the Xi becomes enriched in H2AK119Ub, even though H3K27me3 is not present ^54^, indicating that PRC1-mediated H2AK119Ub can occur on the Xi independently of PRC2. The reverse is also true, however. RING1B ^–/–^ cells that lack the E3 ligase activity responsible for H2AK119Ub show loss of H2AK119Ub from the Xi without affecting H3K27me3 deposition ^55^. Both mutants show reduced levels of their specific histone marks without affecting gene silencing on the Xi. This suggests that there may indeed be several pathways for PRC1 and PRC2 recruitment to the Xi, one of which is direct (PRC1) and the other indirect (PRC2). However, all of the data to date suggest that none of these PRC complexes are sufficient to induce X-chromosome-wide gene silencing during XCI. Rather, Polycomb and its associated chromatin changes are likely to be involved in maintenance, as has been found in other contexts (in mammals and also in flies and worms). This is also supported by studies using inducible Xist transgenes that lack the A-repeat region. These mutants retain the capacity to express Xist and coat the chromosome in *cis*, although to a slightly lesser extent, and they are also able to induce enrichment of H3K27me3 and H2AK119Ub but are not able to induce gene silencing ^56^. Thus, PRC-induced histone modifications are involved in reinforcement and memory mechanisms for gene silencing rather than for initiation.

## RNA methylation as a new player in X-chromosome inactivation

**Figure 1.**
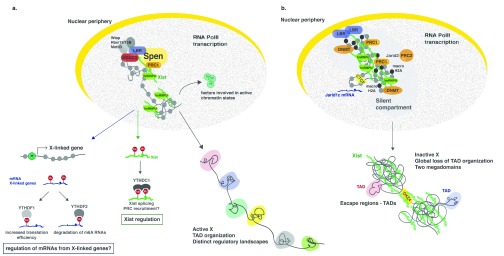
Mechanisms involved in X-chromosome inactivation (XCI). **a**. XCI begins with the expression of Xist RNA, which binds and recruits several protein complexes, such as “Split-ends” (Spen), lamin B receptor (LBR), Wilms tumor 1 associated protein (Wtap)/Rbm15/Rbm15B, Polycomb repressive complex 1 (PRC1), and heterogeneous nuclear ribonucleoprotein U (hnRNPU). At the onset of XCI, the X-linked genes are still transcribed and transcriptional shutdown is starting. Apart from histone deacetylation (via histone deacetylase 3 [HDAC3]), other possible mechanisms for the initiation of gene silencing might involve RNA methylation pathways: either mRNAs from X-linked genes are methylated and targeted for degradation or Xist itself is methylated, possibly stabilizing it, recruiting other protein complexes important for X-linked gene silencing. At this stage, the X chromosome is organized into topologically associating domains (TADs). **b**. With the progression of XCI, the inactive X (Xi) is tethered to the nuclear periphery via Xist interaction with LBR, and the Xi forms a silent compartment devoid of RNA polymerase II (RNA Pol II). Escape genes, like *Jarid1c*, are localized outside this silent compartment, where they are accessible to the transcription machinery. As XCI becomes more established, different chromatin modifications are deposited, such as H2AK119Ub by PRC1, H3K27me3 by PRC2, DNA methylation, and incorporation of the histone variant macroH2A. Upon XCI, the conformation of the Xi dramatically changes, with the loss of TADs and appearance of two megadomains separated by a border containing DXZ4. TAD organization remains only in escape loci of the Xi. CTCF, CCCTC-binding factor; DNMT, DNA methyltransferase 1.

Another protein identified in the Xist pull down experiments was WTAP (Wilms tumor 1 associated protein) ^16, 21^. WTAP is a member of a complex responsible for N ^6^-methyladenosine modification on RNA ^57^, or m ^6^A RNA. RNA methylation is an emerging field, and the many possible functions of this RNA modification on RNA stability, translation, or splicing are still being explored ^58^. However, to date, RNA methylation pathways had never been suspected in XCI. WTAP could have a direct role in Xist RNA methylation, improving its stability, or acting as a guiding mechanism for its target sites ( Figure 1A). Alternatively, WTAP may have a role in Xist-mediated gene silencing by post-transcriptional control. It has previously been reported that mRNAs with m ^6^A groups can be recognized by reader proteins that will be responsible for their function, similar to histone or DNA methylation (reviewed in 59). Two of these readers, YTHDF1 and 2, have opposing roles in transcriptional output: YTHDF1 is correlated with increased translation efficiency, resulting in a positive transcriptional output, but, on the other hand, YTHDF2 seems to reduce the stability of bound m ^6^A mRNAs, targeting them for degradation ^60^. The list of peptides identified in the Xist pull down experiments includes YTHDF3 ^16^, which has been correlated with m ^6^A RNA but has no reported functions to date, and YTHDC1 ^18^, which is involved in exon-inclusion mechanisms in alternative splicing ^61^. Another possible role of m ^6^A RNA is through binding of HNRNPC, which has been reported to affect splicing of target mRNAs and lncRNAs ^62^. HNRNPC was also co-purified with Xist in two of these studies ^17, 18^ and might therefore have a role in Xist splicing and stability. In line with these hypotheses, a recent study reported that Xist is methylated, which is important for its gene silencing function ^63^. In this study, m ^6^A Xist methylation is shown to be recognized by YTHDC1. The role of YTHDC1 in Xist-mediated silencing still remains unclear. The authors propose, based on previously published proteomics studies ^61^ together with protein–protein interaction database analysis ^64^, that YTHDC1 might recruit transcriptional repressors such as PRC1 or PRC2.

In summary, the data obtained so far concerning WTAP’s interaction with Xist appears to point to a role in Xist m ^6^A methylation. However, it is still not clear whether this RNA modification machinery might also act on mRNAs of X-linked genes upon Xist-mediated recruitment. The exploration of RNA modifications could provide exciting new insights into XCI initiation mechanisms.

## Roles for X chromosome conformation and nuclear organization during X-chromosome inactivation

Given the large body of early cytogenetic studies on the Xi describing it as a distinct nuclear compartment (the Barr body) ^65^, a role for nuclear organization has long been proposed in the process of XCI ^66^. Fluorescence microscopy revealed that Xist RNA accumulation during XCI leads to the rapid formation of a repressive compartment, from which RNA Pol II and transcription-associated factors are excluded ^13, 67^. This repressive compartment is largely made of repetitive elements initially ^13^. Genes become relocated into this compartment as they become silenced, while genes that escape remain at the periphery of this Xist RNA domain. The exact interplay between nuclear organization and XCI has remained an open question, however, and whether gene relocation into the Xist RNA compartment is a cause or a consequence of gene silencing is unclear.

Global reorganization of the X chromosome upon Xist RNA coating can also be seen thanks to chromosome conformation capture techniques ^18, 25, 39, 68– 70^. The active X chromosome, just like the autosomes, is organized into contact domains comprising neighboring chromosome regions called topologically associating domains (TADs) as well as into active and inactive compartments ^68, 70, 71^. XCI triggers the loss of TADs and the formation of two megadomains on the Xi ^18, 39, 68, 69^ ( Figure 1B).

How do these chromosomal changes impact on XCI? In one study ^14^, it was shown that deletion of Xist RNA from the Xi in differentiated cells results in the recovery of an active 3D conformation of the X chromosome, even though genes remain silent, presumably due to epigenetic marks such as DNA methylation. This indicates that Xist RNA plays a role in the global organization of X chromosome structure, and a role for Xist in the repulsion of the architectural protein, cohesion, has been proposed ^18^. Although the role of the Xi megadomains is still not clear, the DXZ4 macrosatellite conserved in humans and mice was found to be critical in creating the frontier between them, together with Xist RNA coating ^39, 72^. Deletion of DXZ4 results in the absence of megadomain formation, and surprisingly this does not appear to interfere with XCI onset or maintenance ^39, 72^. Chromatin marks, such as H3K27me3, also remained globally intact upon DXZ4 deletion, except for a region adjacent to the DXZ4 macrosatellite, where there is a loss of H3K27me3 and a gain of H3K9me3 ^72^. Intriguingly, however, the frequency with which some facultative escapees on the X can escape seemed to be slightly affected ^39^, suggesting a potential link between this unusual region and the capacity of some parts of the X to escape.

Finally, in addition to Xist’s role in X chromosome organization, it has also been suggested that it might help to bring the Xi to the nuclear lamina. In fact, nuclear positioning is one of the oldest hypotheses for differential treatment of the two Xs in the same nucleus, by localization at the nuclear lamina ^66^, although both the active and the inactive Xs tend to be fairly peripheral in their nuclear positioning ^73^. Also, the inactive X is often at the nucleolus, not at the periphery ^74– 76^. However, the identification of the lamin B receptor (LBR) as one of Xist’s binding partners retrieved by RAP-MS ^17^ and the fact that its knockdown impairs silencing of specific X-linked genes ( *Gpc4, Atrx, MeCP2, Rbmx*, and *Smc1a*) led to the proposal that LBR may be necessary to tether X-linked regions to the nuclear periphery and that this environment would somehow facilitate Xist RNA-mediated silencing. Impairment of relocalization of the X chromosome might result in impaired X-linked gene silencing owing to a failure to maintain genes within a confined *Xist* silent compartment ^77^. However, the Xist RNA-coated X in LBR mutants is still devoid of RNA Pol II and becomes enriched in PRC2, and the effect on chromosome-wide silencing/escape was not examined ^77^. The exact role of LBR recruitment in XCI and its potential collaboration with other Xist protein partners will undoubtedly warrant future investigation.

## Concluding remarks

The emerging picture from recent studies using diverse molecular, biochemical, and genetic approaches is that XCI involves not just one but multiple gene-silencing mechanisms. The challenge now will be to dissect the specific functions and inter-relationships of these different layers of control. The recently published panel of *Xist* interactors as well as factors identified using genetic screens suggests that even the initiation step of XCI involves a variety of mechanisms. SPEN appears to play a central role and has been reported to interact with HDAC3. This could provide an initiation mechanism via histone deacetylation. On the other hand, WTAP, a member of the RNA methyltransferase complexes, also appears to play a key role in the initiation of XCI by methylation of either *Xist* RNA itself or X-linked mRNAs. Only a few chromatin factors were implicated, maybe surprisingly, and although PRC1 was identified as a direct interactor of Xist, PRC complexes are likely to be brought in by Xist to enable the maintenance of gene silencing rather than its initiation, which may involve histone deacetylation. Finally, recent studies also point to alterations in chromosome organization and nuclear localization being involved in the establishment of a stably inactive state, since Xist RNA triggers a massive reorganization of the Xi in the nucleus. This flurry of new results in the field of XCI has opened up many questions, which will hopefully be answered through the careful dissection of the genetic and molecular pathways involved.
